# Differential diagnosis of gallbladder neoplastic polyps and cholesterol polyps with radiomics of dual modal ultrasound: a pilot study

**DOI:** 10.1186/s12880-023-00982-y

**Published:** 2023-02-06

**Authors:** Hai-xia Yuan, Changyan Wang, Cong-yu Tang, Qi-qin You, Qi Zhang, Wen-ping Wang

**Affiliations:** 1 Department of Ultrasound, Zhongshan Hospital of Fudan University (Qingpu Branch), Shanghai, China; 2 Department of Ultrasound, Zhongshan Hospital of Fudan University, Shanghai, China; 3 Department of Ultrasound, Zhongshan Hospital (Xiamen), Fudan University, Xiamen, Fujian Province China; 4 School of Communication and Information Engineering, Shanghai University, Shanghai, 200444 China; 5 The SMART (Smart Medicine and AI-Based Radiology Technology) Lab, Shanghai Institute for Advanced Communication and Data Science, Shanghai University, Shanghai, China

**Keywords:** Gallbladder polypoid lesion, Superb microvascular imaging, Radiomics

## Abstract

**Purpose:**

To verify whether radiomics techniques based on dual-modality ultrasound consisting of B-mode and superb microvascular imaging (SMI) can improve the accuracy of the differentiation between gallbladder neoplastic polyps and cholesterol polyps.

**Methods:**

A total of 100 patients with 100 pathologically proven gallbladder polypoid lesions were enrolled in this retrospective study. Radiomics features on B-mode ultrasound and SMI of each lesion were extracted. Support vector machine was used to classify adenomas and cholesterol polyps of gallbladder for B-mode, SMI and dual-modality ultrasound, respectively, and the classification results were compared among the three groups.

**Results:**

Six, eight and nine features were extracted for each lesion at B-mode ultrasound, SMI and dual-modality ultrasound, respectively. In dual-modality ultrasound model, the area under the receiver operating characteristic curve (AUC), classification accuracy, sensitivity, specificity, and Youden’s index were 0.850 ± 0.090, 0.828 ± 0.097, 0.892 ± 0.144, 0.803 ± 0.149 and 0.695 ± 0.157, respectively. The AUC and Youden’s index of the dual-modality model were higher than those of the B-mode model (*p* < 0.05). The AUC, accuracy, specificity and Youden’s index of the dual-modality model were higher than those of the SMI model (*p* < 0.05).

**Conclusions:**

Radiomics analysis of the dual-modality ultrasound composed of B-mode and SMI can improve the accuracy of classification between gallbladder neoplastic polyps and cholesterol polyps.

## Introduction

Gallbladder polyps are defined as a general term for a group of diseases that originate in the wall of the gallbladder and protrude restrictively into the lumen [1], also known as polypoid lesions of gallbladder (PLGs). With the popularity of ultrasound, the detection rate of PLGs has increased. Epidemiological studies show that the prevalence of PLGs is about 0.3–12.3% in adults, where gallbladder neoplastic polyps account for only about 5% of PLGs [1, 2]. As the most common gallbladder polyps, cholesterol polyps are foamy cell clusters formed by cholesterol crystals deposited in the gallbladder wall and phagocytosed by macrophages, with the surface covering the mucosal layer of the gallbladder, and no malignant tendency has been reported in the literature [3]. According to the 2019 WHO Classification of Tumors [4], gallbladder neoplastic polyps include pyloric gland adenomas and intracholecystic papillary neoplasms. Gallbladder neoplastic polyps are prone to atypical hyperplasia and may progress to gallbladder cancer, and they are considered as precancerous lesions [5, 6]. Therefore, the accurate differentiation between cholesterol polyps and precancerous gallbladder neoplastic polyps by preoperative imaging is a pressing issue in clinical practice.

Ultrasound is the preferred imaging method for evaluating PLGs, but it is difficult to accurately distinguish cholesterol polyps from gallbladder neoplastic polyps by conventional ultrasound [7, 8]. How to improve the accuracy of ultrasonic identification of gallbladder polyp-like lesions is an urgent clinical problem. Superb microvascular imaging (SMI), as a new modality of ultrasound imaging, has unique advantages in showing the morphology of microvasculature with low flow velocity in the lesion, thereby significantly improving the resolution, sensitivity and specificity of ultrasound diagnosis [9]. The combination of the two ultrasonic modalities, namely conventional ultrasound and SMI, could contribute to more accurate diagnosis of PLG.

Recently, novel imaging technologies based on radiomics (AI) have made rapid advances, where algorithms process medical imaging data sets through hierarchical mathematical models that can learn to use biometrics to detect diagnostic patterns. Zhang et al. [10] established a neoplastic predictive model and evaluated the effectiveness of radiomics in predicting malignancy in patients with gallbladder polyps. A single-center study by Xiang et al. [11] developed and validated a radiomics signature to estimate gallbladder carcinoma recurrence-free survival. At present, the research on the radiomics of gallbladder polyps mainly focuses on the identification of benign and malignant gallbladder polyps and prediction of survival times of patients with gallbladder carcinoma. In this study, we aimed to propose a method based on radiomics to differentiate PLG by leveraging dual-modal ultrasound, namely B-mode ultrasound and SMI, and to further investigate whether it could improve the accuracy of PLG differentiation. We present the following article in accordance with the STARD reporting checklist.

## Materials and methods

### Patient clinical data

This retrospective study was approved by the Institutional Ethics Committee at our institution (B2022-187R) for the retrospective review of images and patients’ medical records, and the need for informed consent was waived for the retrospective design. In total, 100 patients with 100 gallbladder polyps between January 2019 and December 2021 were included (43 males and 57 females; aged 21 to 58, mean ± standard deviation, 35.1 ± 7.5 years). All the patients underwent preoperative conventional ultrasound and SMI before cholecystectomy. Pathological examination revealed that the diameters of the lesions were 0.8 to 2.8 cm and the detailed categories of the lesions were: cholesterol polyps (71 lesions), tubular adenoma (11 lesions), tubular adenoma with moderate or severe atypical hyperplasia (12 lesions), and villous adenoma with focal carcinoma (6 lesions). The clinical and lesion characteristics are summarized in Table 1. The difference in age between the gallbladder neoplastic polyps and cholesterol polyps groups has been tested using independent sample t-tests, and the difference in constituent ratios of sex between the gallbladder neoplastic polyps and cholesterol polyps groups has been tested using χ^2^ test.

**Table 1:** Clinical data of gallbladder cholesterol polyps and gallbladder neoplastic polyps

Parameter	Gallbladder cholesterol polyps (n = 71)	Gallbladder neoplastic polyps (n = 29)	*p-*value
Age (y)			
Male	35 (30–53)	41 (35–50)	0.43
Female	30 (21–56)	37 (31–58)	0.61
Sex*			0.51
Male	32	11	/
Female	39	18	/
Lesion size (mm)	13 (8–21)	17 (8–28)	0.04
Number of polyp (solitary/multiple)*	64/7	27/2	0.35
Shape (regular/irregular)*	20/51	9/20	0.58
Basal structure (sessile/pedunculated)*	55/16	27/2	< 0.001
Internal echogenicity (hyperechoic/isoechoic/hypoechoic)*	21/27/23	6/16/7	0.12
Vascularity (yes/no)*	38/33	26/3	< 0.001

Numbers in parentheses are a range

*Data are numbers of patients

### Instrumentation and image acquisition

A color Doppler ultrasound system (Canon Aplio500, Japan) equipped with a 3.5–5.0 MHz transducer was used in this study. After fasting for more than eight hours, the patients underwent ultrasonic examination in the left lateral position. The imaging process consisted of two phases. In the first phase, the doctor would use the probe to scan the gallbladder area of each patient, then locally enlarge the lesion area and instruct the patient to hold his/her breath to show the largest section of the lesion, then record the maximum diameter at the right time.

In the second phase, the color Doppler ultrasound was used to observe internal vascularity of the lesion and the monochrome SMI (mSMI) was performed [12]. Sections with abundant vascularity inside or at the edge of lesion were probed. Patients were instructed to hold their breath, meanwhile, the doctor continually adjusted the gain and the size of the sampling frame until the small vessels were just detected and the entire lesion and its surrounding 1 cm area was included in the sampling frame as much as possible. The velocity scale was set to less than 3 cm/s. If no vascularity could be detected in the lesion, the maximum diameter section was selected for examination. The imaging system provided dual-modal visualization in a full screen, where the left part was a grayscale B-mode image, and the right part was an SMI image. The static images of B-mode and SMI were selected and saved for radiologists’ visual evaluation of gallbladder polyps.

### Radiomics analysis of dual-modality ultrasound image

#### Framework for the identification of gallbladder polyps

**Fig. 1:**
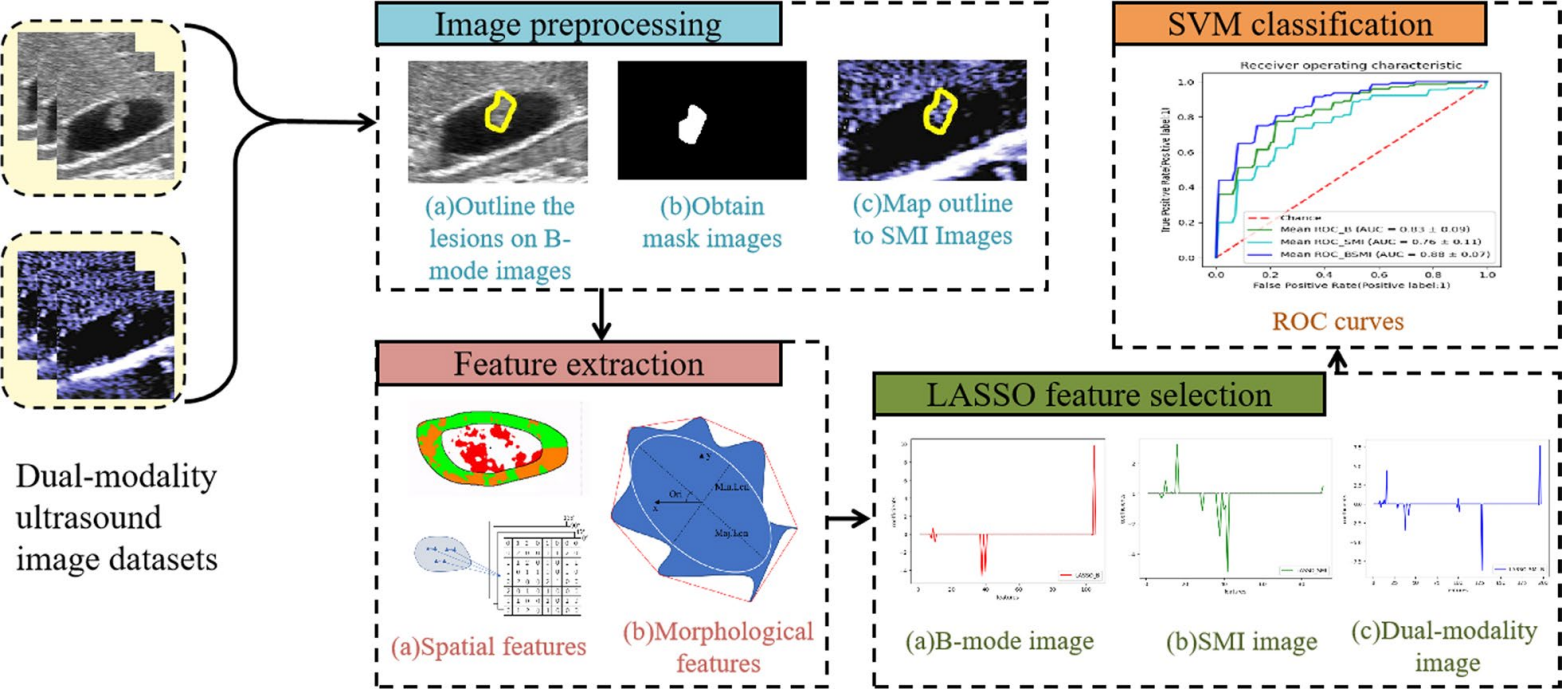
Radiomics analysis process of gallbladder polyps in dual-modality ultrasound images consisting of B-mode and SMI

The process of radiomics analysis based on dual-modality ultrasound images mainly included the following steps [13, 14]. (1) Image preprocessing: outlining the lesions on the dual-modality images, and then binarizing B-mode ultrasound images to obtain binary mask images; (2) Feature extraction: extracting spatial features from dual-modal images and morphological features from B-mode ultrasound images; (3) Feature selection: selecting features with interclass correlation coefficient (ICC) and the least absolute shrinkage and selection operator (LASSO) to reduce dimension of high-dimensional feature information; (4) Classification: using the support vector machine (SVM) algorithm to classify gallbladder polyps. The flowchart is shown in Fig. 1.

#### Image preprocessing

**Fig. 2:**
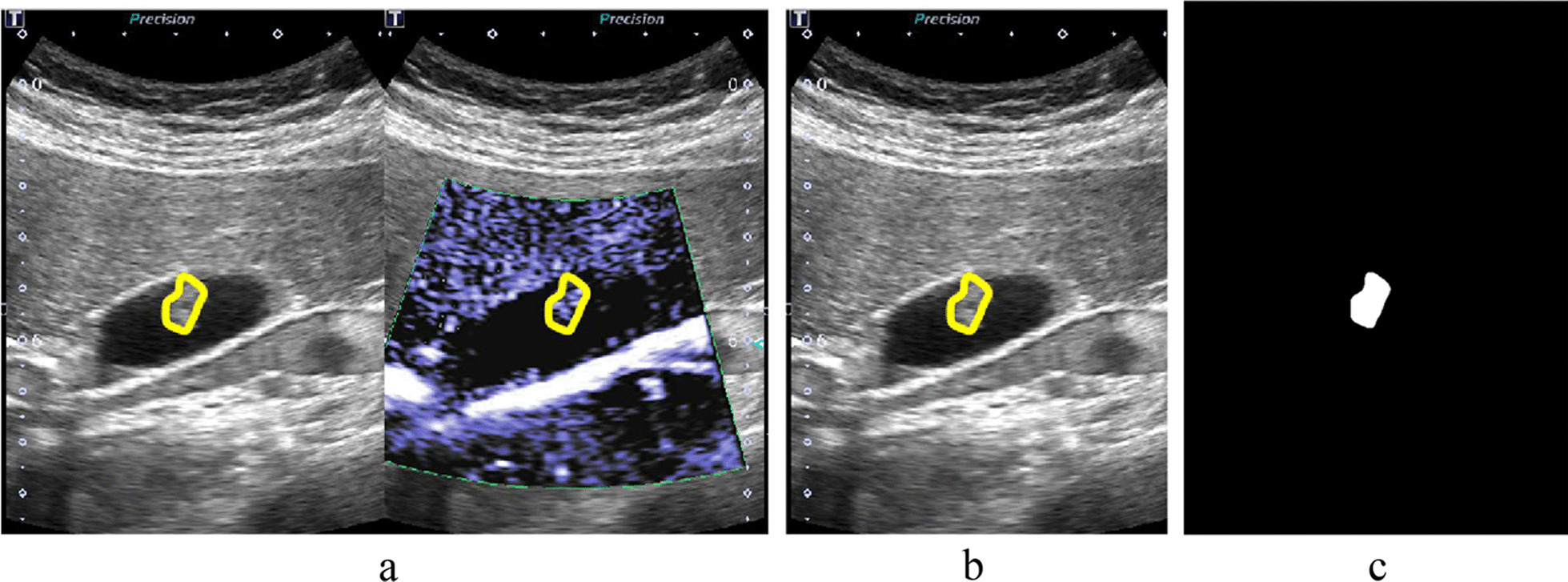
Dual-modality ultrasound image preprocessing. **a** Dual-modality images marked with lesion outlines. **b** B-mode ultrasound image marked with lesion outline. **c** Mask image of B-mode ultrasound

It was necessary to determine the lesion areas on the dual-modality images before quantitative analysis of ultrasound images. Two radiologists performed lesion delineation three times (one of them performed lesion delineation twice) to test the features’ stability by calculating the inter- and intra-observer variability of the features. First, the edge of each lesion was circled on the original B-mode ultrasound images with drawing software [15], as shown in Fig. 2b. Second, the B-mode images were binarized by thresholding segmentation, then the outline was filled with white and the rest was set to black to obtain the mask images, as shown in Fig. 2c. Finally, the location of the lesion and the morphological information of the outline in the B-mode ultrasound images were mapped to the SMI images, as shown in Fig. 2a.

#### Feature extraction

##### Spatial feature extraction

According to the original dual-modality ultrasound images and the mask images of the B-mode ultrasound images, the spatial features were extracted, including the first-order statistics features, the gray level co-occurrence matrix (GLCM) texture features and binary texture features [13, 14].

The first-order statistics features included the mean (IMean), the median (IMedian), standard deviation (SD), coefficient of variation (CoV), histogram entropy, skewness, kurtosis of the pixels within the lesion. Besides, RImedian (RImean) was defined as the corresponding ratio of the median (mean) of the pixels within the lesion and within the reference area (the rectangular area expanding outwards from the lesion).

GLCM was an important technique for texture analysis [16], which represented characteristics of the intensity distribution and the respective distance of intensity levels in the original image. In this study, the GLCM texture features included four types: energy (Ener), contrast (Cont), entropy (Entr) and homogeneity (Homo). Each type of GLCM features were constructed for different values of offset *d*, which was an integer between 1 and 15 pixels. Therefore, each type of GLCM feature included 15 texture features, and 60 GLCM texture features were extracted for each lesion.

Binary texture features were extracted based on the mask images. Features included: the area ratio (AR), which denoted the ratio of the high-intensity area to the whole lesion area; the center deviation degree (CDD), which characterized the normalized distance between each pixel point in the high intensity area of the lesion and the center point of the lesion; the dispersion degree (DD), which characterized the mean of normalized Euclidean distance between each pixel point in the high-intensity area of the lesion and the center point of the high-intensity area.

##### Morphological feature extraction

Since the morphological features of lesions in each modality were consistent, we only extracted morphological features based on B-mode ultrasound images [15]. The morphological features included: the area of the lesion (Area), the area of minimum convex polygon corresponding to the lesion (C.area), and the long axis length (Maj.Len), etc.

#### Feature selection

We used ICC and the LASSO to select extracted features based on dual-modality images. By reducing the dimension of the extracted features and removing redundant information and irrelevant features [17–21], a subset of features useful for diagnosis of PLGs were selected.

Firstly, two investigators independently outlined the ROIs of all the images. And then we extracted the radiomic features, calculated the inter- and intra-observer ICC values of the radiomic features, and then selected the features with inter-observer ICC values greater than 0.6 for LASSO regression analysis. Before LASSO feature selection, the features were normalized to eliminate the effect of extreme values and different magnitudes. Secondly, according to the features extracted from the B-mode ultrasound images, SMI images and the combined dual-modality images, the most suitable threshold was selected at which the model prediction error was minimized. Finally, the three sets of features were selected by LASSO method respectively, so as to filter out three subsets of features after dimension reduction from the three modal images.

#### SVM classification

**Fig. 3:**
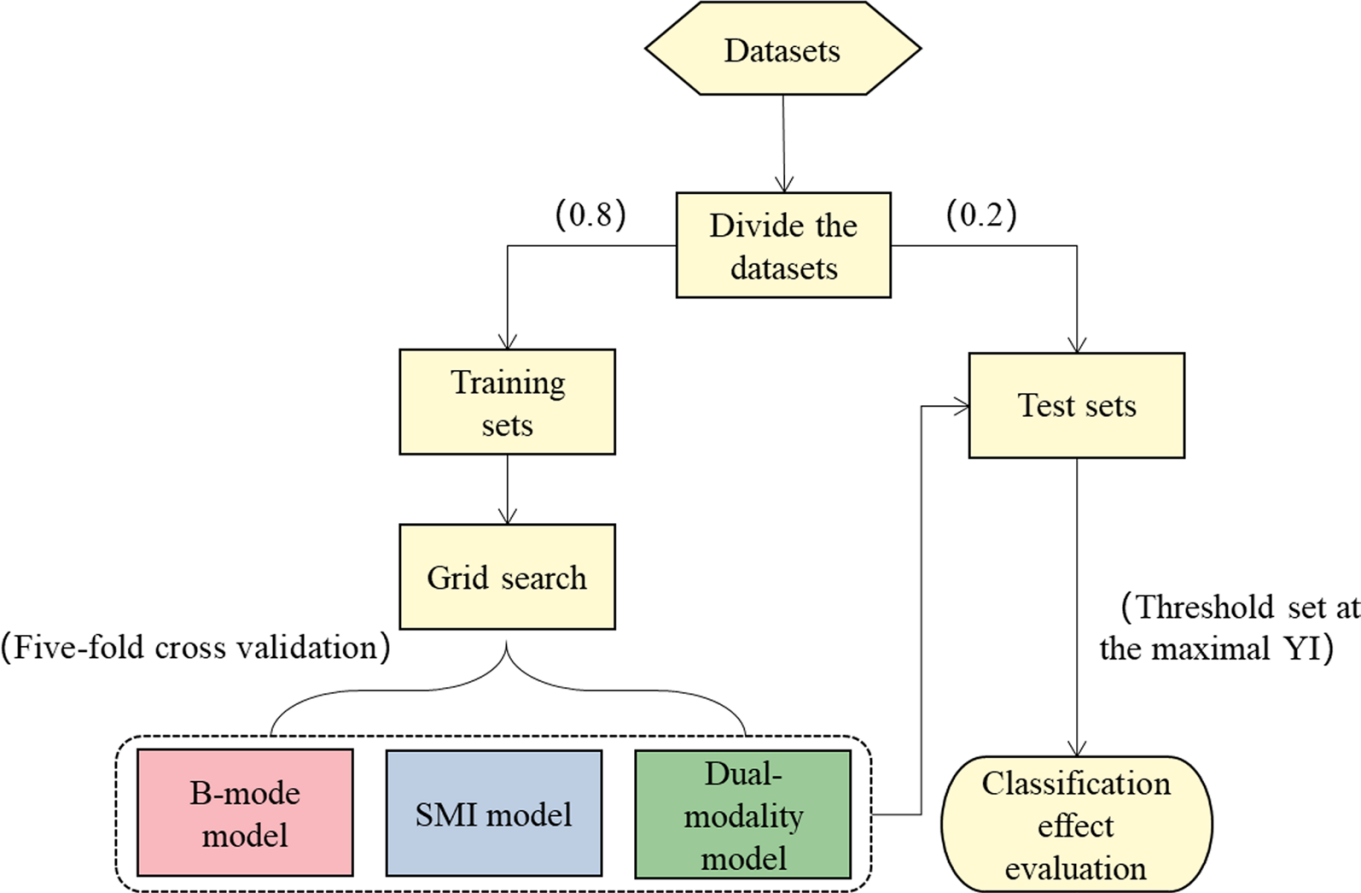
SVM classification algorithm flowchart

The dual-modality ultrasound images of gallbladder polyps collected in this experiment belong to a small sample set. Considering the high applicability of the SVM to small sample sets, we used this algorithm to classify gallbladder polyps [22]. In this paper, the filtered feature subsets of the three groups of modalities were used as the original datasets for SVM classification. As shown in Fig. 3, first, the three groups of feature subsets were divided into training sets and test sets according to the ratio of 8:2. The classification model was trained on the three training sets respectively. Then the kernel function parameters of the SVM model were adjusted by grid search, and the five-fold cross validation method was used to obtain the optimal model with the smallest generalization error respectively. The three optimal models, namely as B-mode model, SMI model and dual-modality model, were applied to their corresponding test sets for classification. Five-fold cross validation was performed five times. Finally, five classification evaluation indicators were used in this paper to evaluate the classification effects of the three groups, the indicators were: the area under the receiver operating characteristic curve (AUC), accuracy (ACC), sensitive (SEN), specificity (SPE) and Youden’s index (YI) [13, 23].

### Statistical analysis

Both the paired t-test and the Wilcoxon signed rank test are statistical test methods used to test whether the mean difference between paired measurements is 0 [19]. The paired t-test requires that the differences between paired sample data satisfy a normal distribution or approximate normal distribution. If the difference of each pair of sample data satisfies normal distribution, the paired t-test can be used. If the difference is seriously skewed distribution, the Wilcoxon signed rank test can be used. In order to further explore the difference between the dual-modality model and the single-modality model in the classification of gallbladder polyps, a paired design was carried out for the B-mode model and the dual-modality model, the SMI model and the dual-modality model, respectively.

## Results

### Results on B-mode images

After feature extraction, we obtained 91 spatial features and 15 morphological features from B-mode ultrasound images. There were 38 features with the inter-observer ICC values above 0.6 and 90 features with the intra-observer ICC values above 0.6. Through feature selection, a total of 6 spatial features were retained. The classification results of SVM algorithm are shown in Table 3.

### Results on SMI images

In this paper, 91 spatial features were extracted from SMI images. There were 26 features with the inter-observer ICC values above 0.6 and 91 features with the intra-observer ICC values above 0.6. After feature selection, a total of 8 spatial features were retained. The classification results of SVM algorithm are shown in Table 3.

### Results on dual-modality images

**Fig. 4:**
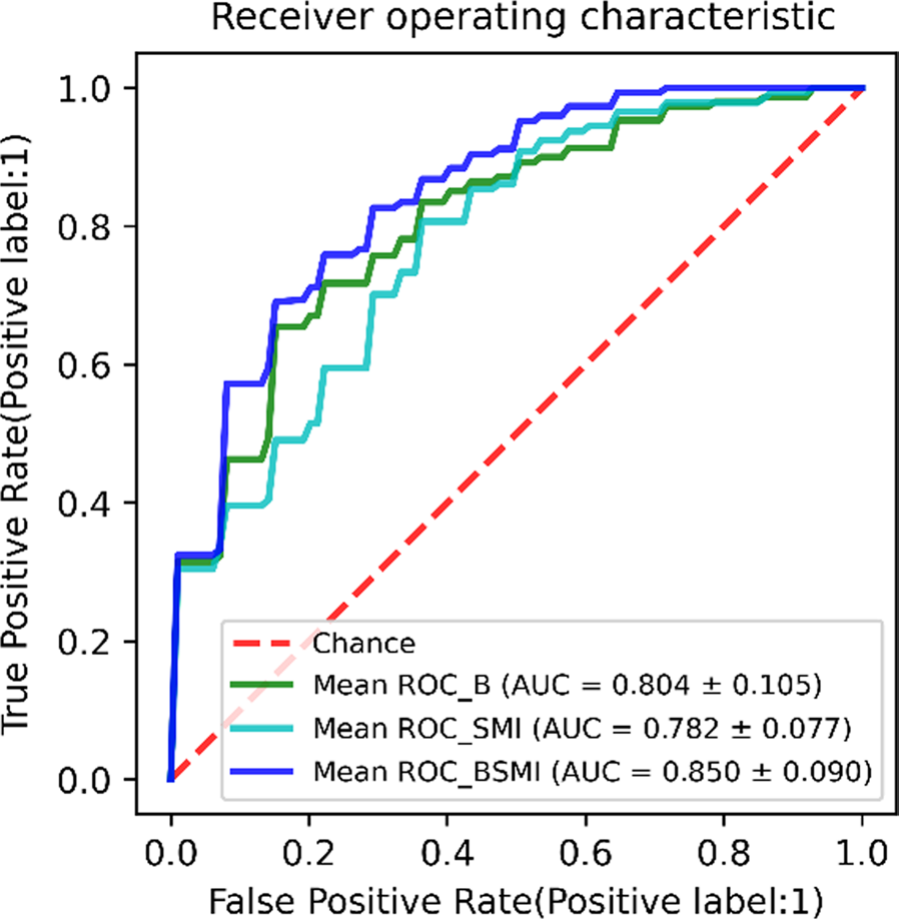
ROC curves of different models

**Fig. 5:**
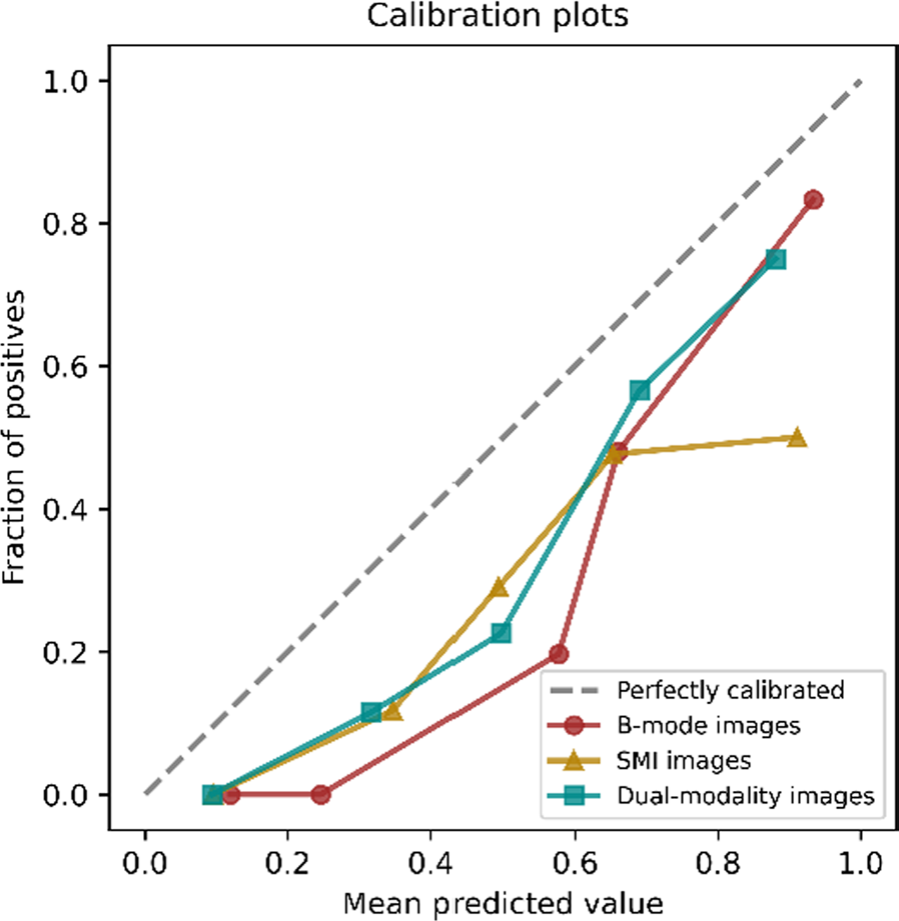
Calibration curves of different models

The lesion features of the dual-modality images contained 182 spatial features and 15 morphological features, including 91 spatial features and 15 morphological features from B-mode ultrasound images and 91 spatial features from SMI images. There were 64 features with the inter-observer ICC values above 0.6 and 181 features with the intra-observer ICC values above 0.6. Feature selection was used on the 197 features of the dual-modality images, and 9 spatial features were retained. The feature selection results of different modalities are shown in Table 2. The classification results of SVM algorithm are shown in Table 3. We drew the receiver operating characteristic (ROC) curves of different modes according to the classification indicators [15], as shown in Fig. 4. We drew the calibration curves of different models, as shown in Fig. 5. Calibration curves depicted the calibration of each model in terms of the agreement between the predicted risks of gallbladder neoplastic polyps and observed outcomes of gallbladder neoplastic polyps. The y-axis represented the actual gallbladder neoplastic polyp rate. The x-axis represented the predicted gallbladder neoplastic polyp risk. The diagonal dotted line represented a perfect prediction by an ideal model. Three solid lines represented the performance of three different models, of which a closer fit to the diagonal dotted line represented a better prediction. Calibration curves demonstrated that the dual-modality model had better agreement between prediction and observation than the single-modality models.

**Table 2:** Quantitative features automatically selected from different modality images

B-mode ultrasound image	SMI image	Dual-modality image
IMean	Imean	IMean_SMI
IMedian	Imedian	Std_I_SMI
Std_I	Std_I	Cont8_SMI
RAR3	RImean	Cont11_SMI
Cont7	AR_O2	Cont15_SMI
Cont15	Cont8	IMean_B
	Cont11	IMedian_B
	Cont15	Std_I_B
		Cont7 _B

**Table 3:** Classification results

Data sets	Model	AUC	ACC	SEN	SPE	YI
Training sets	B-mode	0.874 ± 0.033	0.832 ± 0.056	0.792 ± 0.096	0.851 ± 0.095	0.636 ± 0.090
	SMI	0.872 ± 0.080	0.814 ± 0.047	0.880 ± 0.140	0.793 ± 0.076	0.668 ± 0.123
	Dual-modality	0.899 ± 0.036	0.861 ± 0.046	0.852 ± 0.092	0.864 ± 0.064	0.716 ± 0.097
Test sets	B-mode	0.804 ± 0.105	0.802 ± 0.084	0.831 ± 0.152	0.792 ± 0.137	0.622 ± 0.139
	SMI	0.782 ± 0.077	0.756 ± 0.092	0.856 ± 0.158	0.718 ± 0.166	0.574 ± 0.125
	Dual-modality	0.850 ± 0.090	0.828 ± 0.097	0.892 ± 0.144	0.803 ± 0.149	0.695 ± 0.157

Finally, the statistical analysis results of the above three classification experiments were obtained. The paired t-test and Wilcoxon signed rank test were used on the dual-modality model with the B-mode model and the SMI model, respectively. There was statistical difference in the AUC, YI between B-mode model and dual-modality model (AUC’s difference is 0.045, 95% CI − 0.003–0.093, *p* = 0.045; YI’s difference is 0.073, 95% CI 0.001–0.145), *p* = 0.039). There was statistical difference in SPE between SMI model and dual-modality (SPE’s difference is 0.085, 95% CI 0.016–0.154, *p* = 0.018). And there was a significantly statistical difference in AUC, ACC and YI between these two groups (AUC’s difference is 0.068, 95% CI 0.039–0.097, *p* < 0.001; ACC’s difference is 0.072, 95% CI 0.037–0.107, *p* < 0.001; YI’s difference is 0.121, 95% CI 0.068–0.174), *p* < 0.001). The statistical test results are shown in Table 4.

**Table 4:** The statistical difference between the dual-modality and single-modality models

Indicator	Model	Mean ± Standard deviation	Difference and 95% CI	*p-*value
AUC	B-mode	0.804 ± 0.105	0.045 (− 0.003–0.093)	0.045^*^
	SMI	0.782 ± 0.077	0.068 (0.039–0.097)	< 0.001
	Dual-modality	0.850 ± 0.090	–	–
ACC	B-mode	0.802 ± 0.084	0.026 (− 0.020–0.072)	0.257
	SMI	0.756 ± 0.092	0.072 (0.037–0.107)	< 0.001
	Dual-modality	0.828 ± 0.097	–	–
SEN	B-mode	0.831 ± 0.152	0.061 (− 0.022–0.144)	0.140
	SMI	0.856 ± 0.158	0.036 (-0.045–0.117)	0.264^*^
	Dual-modality	0.892 ± 0.144	–	–
SPE	B-mode	0.792 ± 0.137	0.012 (− 0.066–0.090)	0.761
	SMI	0.718 ± 0.166	0.085 (0.016–0.154)	0.018
	Dual-modality	0.803 ± 0.149	–	–
YI	B-mode	0.622 ± 0.139	0.073 (0.001–0.145)	0.039^*^
	SMI	0.574 ± 0.125	0.121 (0.068–0.174)	< 0.001
	Dual-modality	0.695 ± 0.157	–	–

*The *p*-value is the result of the Wilcoxon signed rank test due to the non-normal distribution, the remaining *p*-values are the results of the paired t-test

## Discussion

PLGs are mostly detected during physical checkup with increasing incidence. For gallbladder neoplastic polyps, since the prognosis is closely related to the stage of gallbladder cancer at the time of surgery, timely cholecystectomy is important. The five-year survival rate of gallbladder cancer after surgery is 2–80%. The five-year survival rate for in situ gallbladder cancer can reach 80%, while dropping to 8% when there is lymph node metastasis, and the rate of stage 4b gallbladder cancer is only 2% [24]. Therefore, early detection of gallbladder cancer and precancerous lesions and early intervention are important to improve the survival rate of patients.

Ultrasound has been recognized as the first choice of imaging examination for the screening and follow-up of gallbladder polyps. However, it is difficult to identify gallbladder neoplastic polyps or cholesterol polyps solely from the echogenicity, morphology, or vascularity characteristics of the lesion [25, 26]. In Table 1 of this study, although the polyp diameter, pedicle and blood flow are statistically different between the gallbladder neoplastic polyp group and cholesterol polyp group, the diagnostic accuracy is low based on these indicators. Accordingly, Domestic and international scientific guidelines over the years recommend cholecystectomy for gallbladder polyps larger than 1 cm in diameter [27]. However, the guidelines for gallbladder polyps have resulted in a large number of unnecessary cholecystectomy, which has been questioned by many scholars [28, 29]. Therefore, there is an urgent need for a new method with high accuracy and reproducibility to accurately identify gallbladder neoplastic polyps and cholesterol polyps.

The application of artificial intelligence in medical imaging is one of the hot spots in medical research. Based on the big data analysis of computer, we can obtain numerous objective image feature data with a resolution far beyond the human eye [16, 30–32]. In our previous study, we extracted spatial and morphological features of single-modality gray-scale ultrasound. Our study reported that adenomas polyps have a more uniform pixel distribution, with a relatively smaller proportion of hyperechoic areas inside the polyps, and adenomas polyps are larger and more irregular in morphology. These are closely related to the pathophysiological features of gallbladder neoplastic polyps and cholesterol polyps [3, 6].

In this study, we extracted dual-modality ultrasound image datasets of gray-scale ultrasound and SMI. SMI technique is a method to evaluate tissue microvascular perfusion, and it can detect low speed flow signal without contrast agent, thus giving us more diagnostic information. In the process of radiomics analysis in this paper, the SVM model using dual-modality images’ features had the best discriminative ability, and its AUC, ACC, SEN, SPE and YI all reached the best level. There were statistical differences in the AUC, YI between the dual-modality images and the B-mode images. And there were statistical differences in the AUC, ACC, SPE and YI between the dual-modality images and SMI images. It could be seen that the calibration curve (Fig. 5) of the dual-modality model was closer to the diagonal dotted line corresponding to the perfect prediction model than the single-modality models, which indicated that the dual-modality model had better prediction performance. In conclusion, the dual-modality images combined with B-mode images and SMI images have the potential to improve the accuracy of classification of PLGs. In future clinical practice, the dual-modality radiomics features will be extracted, and the classification model based on the dual-modality radiomics features will help to accurately and early identify the gallbladder neoplastic polyps from cholesterol polyps.

Our study also had some limitations. This study was our preliminary attempt to obtain dual-modality ultrasound images’ parameters as well as apply radiomics technology to identify gallbladder neoplastic polyps and cholesterol polyps of gallbladder. The sample size of this study was small, it will be expanded for deep learning in the follow-up [33]. Moreover, this study was a single-center study, and a prospective multicenter study with a large sample size need to be conducted for further validation in the future. The results in this paper are preliminary and guarantee further robust studies in the future.

## Conclusions

In conclusion, with the analysis of radiomics, the dual-modality ultrasound combining B-mode ultrasound and SMI showed an excellent classification accuracy for gallbladder neoplastic polyps and cholesterol polyps of gallbladder. Our model has high sensitivity and specificity at differentiating gallbladder polyps, which means that it could be potentially used in clinical practice to avoid unnecessary cholecystectomies and missing diagnosis of gallbladder neoplastic polyps.

## Data Availability

The datasets used and/or analyzed during the current study are available from the corresponding author on reasonable request.

## Abbreviations

SMI: Superb microvascular imaging
PLG: Polypoid lesion of gallbladder
LASSO: Least absolute shrinkage and selection operator
GLCM: Gray level co-occurrence matrix
AUC: Area under the receiver operating characteristic curve
ACC: Accuracy
SEN: Sensitive
SPE: Specificity
YI: Youden’s index
